# Importance of cerebrospinal fluid analysis in the era of McDonald 2010 criteria: a German–Austrian retrospective multicenter study in patients with a clinically isolated syndrome

**DOI:** 10.1007/s00415-016-8302-1

**Published:** 2016-10-11

**Authors:** André M. Huss, Steffen Halbgebauer, Patrick Öckl, Corinna Trebst, Annette Spreer, Nadja Borisow, Andrea Harrer, Isabel Brecht, Bettina Balint, Oliver Stich, Sabine Schlegel, Nele Retzlaff, Alexander Winkelmann, Romy Roesler, Florian Lauda, Özlem Yildiz, Elke Voß, Rainer Muche, Sebastian Rauer, Florian Then Bergh, Markus Otto, Friedemann Paul, Brigitte Wildemann, Jörg Kraus, Klemens Ruprecht, Martin Stangel, Mathias Buttmann, Uwe K. Zettl, Hayrettin Tumani

**Affiliations:** 1 Department of Neurology, University of Ulm, Oberer Eselsberg 45, 89081 Ulm, Germany; 2 Hannover Medical School, Hannover, Germany; 3 University of Göttingen, Göttingen, Germany; 4 NeuroCure Clinical Research Center, Charité-Universitätsmedizin Berlin, Berlin, Germany; 5 Department of Neurology and Clinical and Experimental Multiple Sclerosis Research Center, Charité-Universitätsmedizin Berlin, Berlin, Germany; 6 Experimental and Clinical Research Center, Max Delbrueck Center for Molecular Medicine, Charité-Universitätsmedizin Berlin, Berlin, Germany; 7 Department of Neurology, Paracelsus Medical University Salzburg, Salzburg, Austria; 8 University of Würzburg, Würzburg, Germany; 9 University of Heidelberg, Heidelberg, Germany; 10 UCL Institute of Neurology, London, UK; 11 University of Freiburg, Freiburg, Germany; 12 Neuroimmunological Section, Department of Neurology, University of Rostock, Rostock, Germany; 13 University of Leipzig, Leipzig, Germany; 14 Department of Laboratory Medicine, Paracelsus Medical University Salzburg, Salzburg, Austria; 15 University of Düsseldorf, Düsseldorf, Germany

**Keywords:** OCB, CSF, Multiple sclerosis, Biomarker

## Abstract

The majority of patients presenting with a first clinical symptom suggestive of multiple sclerosis (MS) do not fulfill the MRI criteria for dissemination in space and time according to the 2010 revision of the McDonald diagnostic criteria for MS and are thus classified as clinically isolated syndrome (CIS). To re-evaluate the utility of cerebrospinal fluid (CSF) analysis in the context of the revised McDonald criteria from 2010, we conducted a retrospective multicenter study aimed at determining the prevalence and predictive value of oligoclonal IgG bands (OCBs) in patients with CIS. Patients were recruited from ten specialized MS centers in Germany and Austria. We collected data from 406 patients; at disease onset, 44/406 (11 %) fulfilled the McDonald 2010 criteria for MS. Intrathecal IgG OCBs were detected in 310/362 (86 %) of CIS patients. Those patients were twice as likely to convert to MS according to McDonald 2010 criteria as OCB-negative individuals (hazard ratio = 2.1, *p* = 0.0014) and in a shorter time period of 25 months (95 % CI 21–34) compared to 47 months in OCB-negative individuals (95 % CI 36–85). In patients without brain lesions at first attack and presence of intrathecal OCBs (30/44), conversion rate to MS was 60 % (18/30), whereas it was only 21 % (3/14) in those without OCBs. Our data confirm that in patients with CIS the risk of conversion to MS substantially increases if OCBs are present at onset. CSF analysis definitely helps to evaluate the prognosis in patients who do not have MS according to the revised McDonald criteria.

## Introduction

Multiple sclerosis (MS) is a chronic inflammatory disease mainly characterized by demyelination and axonal loss [1]. A formal diagnosis of MS is based on clinical and radiological findings with an increasing role of MRI examinations as established in the 2010 revision of the so-called McDonald diagnostic criteria [2]. Here, cerebrospinal fluid (CSF) analysis, and in particular the detection of intrathecal IgG oligoclonal bands (OCBs), is a supportive criterion for a diagnosis of primary progressive multiple sclerosis (PPMS) but not in the more common relapsing–remitting form (RRMS) [2]. Nevertheless, the importance of OCBs, especially in the context of differential diagnosis and misdiagnosis in MS, is shown in several studies [3–7]. Most often, the disease starts with a single clinical attack which is termed clinically isolated syndrome (CIS) when the MRI criteria of dissemination in space and time are not fulfilled [1, 8]. Several biomarkers allowing the prediction of conversion from CIS to clinically definite multiple sclerosis (CDMS) have been suggested [9–13]: Besides cerebrospinal demyelinating MRI lesions, especially OCBs restricted to the CSF of CIS patients are associated with a higher risk for conversion to CDMS independent of the baseline MRI results [14, 15]. Additionally, positive OCB predicts CDMS in children with optic neuritis [16]. However, most of these CSF studies were conducted in the context of previous diagnostic criteria, i.e., McDonald criteria 2005.

In this study, we aimed to evaluate the prevalence and predictive value of OCBs in the context of the revised McDonald criteria 2010 [2] and therefore retrospectively analyzed 406 patients with a first presentation suggestive of MS from ten centers in Germany and Austria. We compared MRI and OCB findings as well as the disease course and conversion to MS according to McDonald 2010 criteria over a follow-up time of up to 12 years (median 32 months).

## Materials and methods

### Participants and inclusion criteria

In total, 406 patients with a first manifestation suggestive of MS and for whom sufficient baseline CSF and MRI data were available as well as clinical and radiological follow-up were included. MRI data were considered sufficient if theses enabled to classify according to the Swanton criteria for dissemination in space [17], Montalban criteria for dissemination in time [18] and the revised McDonald 2010 criteria for RRMS [2]. CIS was defined as a first clinical event suggestive of MS not yet meeting the revised McDonald 2010 criteria for RRMS. Patients were included irrespective of the number of T2 hyperintense lesions on cerebral MRI at baseline, i.e., also patients without T2 hyperintense lesions on cerebral MRI were included in the study. Patients who already fulfilled the revised McDonald criteria 2010 for a diagnosis of MS were not taken into account for the evaluation of predictive factors (MRI and OCB findings) concerning conversion to definite MS according to McDonald 2010 criteria.

CSF and serum samples were analyzed for routine workup in the local centers according to international recommendations on standards for CSF analysis [19].

### Data collection

The diagnostic workup, including MRI, CSF and clinical assessment, was performed in each participating center. Data were collected retrospectively with the help of an Excel spreadsheet. The number and localization of T2 and gadolinium-enhancing (GD+) lesions on MRIs were evaluated at each participating center. Detection of intrathecal immunoglobulin (IgG) OCBs was performed using isoelectric focusing followed by immunoblotting, immunofixation or rarely silver staining. Additional CSF data like leukocyte count, albumin CSF-to-serum quotient (QAlb), IgG CSF-to-serum quotient (QIgG) and demographic data were provided by each center.

### Statistical analyses

Absolute and relative frequencies are given for discrete variables, and median and interquartile range for continuous variables. Differences between CIS–CIS and CIS-MS were analyzed by Chi Square test or Mann–Whitney *U* test on a univariate basis in an exploratory sense. Kaplan–Meier surviving analysis was performed to assess conversion to definite MS and hazard ratios were calculated by Cox proportional hazard model. *p* values below 0.05 were considered to be significant. Sensitivity was calculated as (true positive/[true positive + false negative]), and specificity was calculated as (true negative/[true negative + false positive]). The positive predictive value (PPV) was calculated as (true positive/[true positive + false positive]), and the negative predictive value (NPV) as (true negative/[true negative + false negative]). For all diagnostic values, the exact 95 % confidence intervals were given.

## Results

**Fig. 1:**
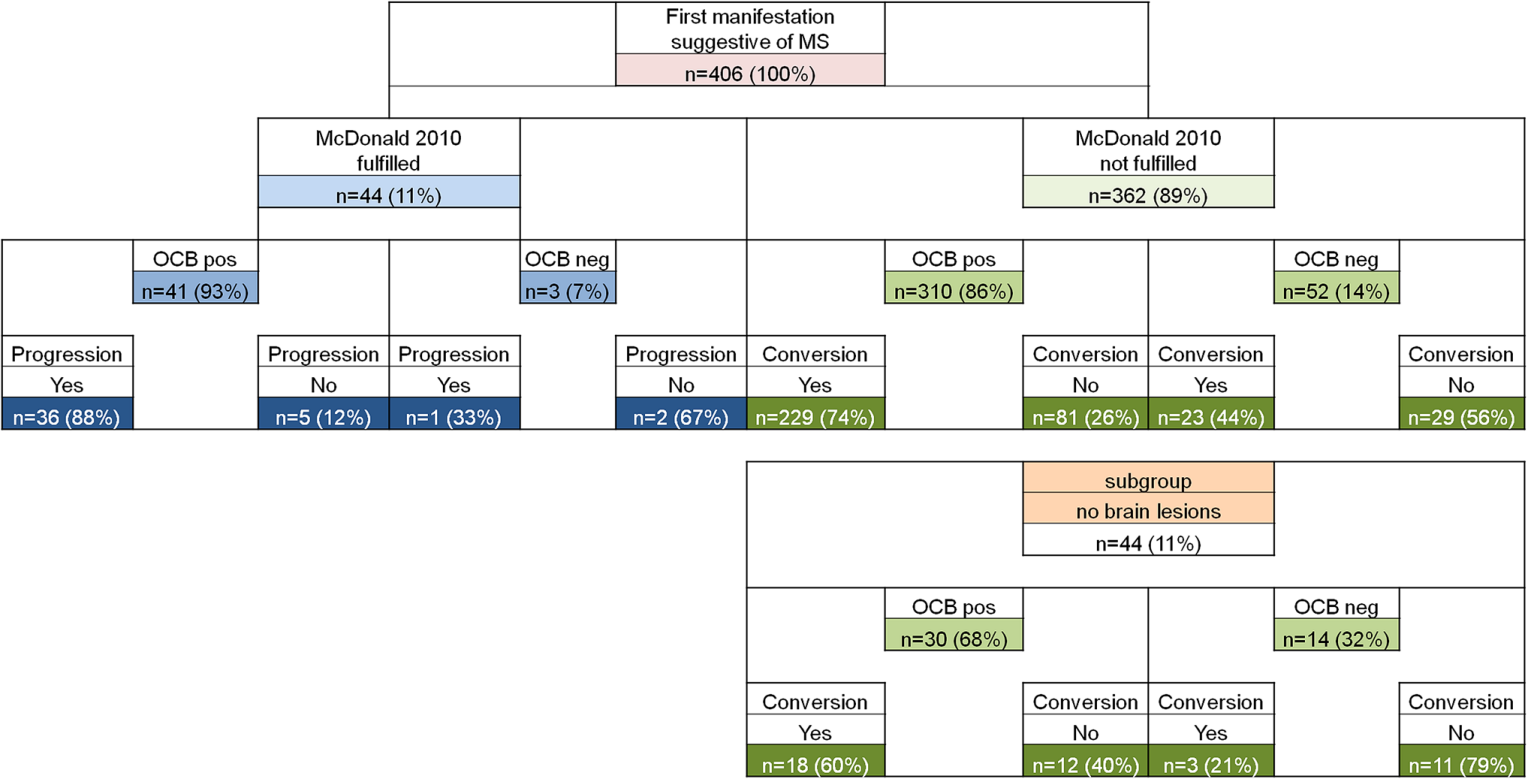
MRI (according to the revised McDonald criteria 2010) and OCB characteristics of all CIS patients. *MS* multiple sclerosis, *MRI* magnetic resonance imaging, *OCB* oligoclonal bands, *CIS* clinically isolated syndrome, *Progression* follow-up disease activity by clinical or MRI signs, *Conversion* fulfillment of the revised McDonald criteria 2010 in the follow-up time, *n.d.*  not determined

We collected data from 406 patients, 277 (68 %) of whom were female. The mean age at clinical onset was 37 years (SD ± 12). At disease onset, 44/406 (11 %) patients fulfilled the McDonald 2010 criteria for MS [2], 137/406 (34 %) the Swanton criteria (dissemination in space) [17], 87/406 (21 %) the Montalban (dissemination in time) criteria [18], and 44/406 (11 %) had no brain lesions. Intrathecal IgG OCBs were detected in 351/406 (86 %) and in 310/362 (86 %) CIS patients. 229/310 (74 %) converted to MS clinically or on MRI according to the McDonald 2010 criteria during the follow-up period of up to 154 months (median 32 months). All patient characteristics, as well as MRI and OCB findings, are summarized in Table 1 and Fig. 1.

**Table 1:** Overview on demographics and clinical data

	All patients (*n* = 406)	CIS patients (*n* = 362)		*p* values CIS patients OCB pos. vs. neg.
		OCB positive (*n* = 310)	OCB negative (*n* = 52)	
Age (years)	36 (27–46)	36 (27–46)	39 (28–44)	0.59
Females	277 (68 %)	215 (69 %)	31 (60 %)	0.16
Follow-up (months)	32 (15–50)	33 (16–50)	24 (13–48)	0.16
McDonald 2010	44 (11 %)	–	–	–
Swanton	137 (34 %)	82 (26 %)	11 (21 %)	0.42
Montalban	87 (21 %)	35 (11 %)	8 (15 %)	0.40
Conversion to definite MS	–	229 (74 %)	23 (44 %)	<0.0001
Cell count (n/µl)	6 (3–12)	6 (3–12)	2 (1–3)	<0.0001
OCB positive	351 (86 %)	310 (86 %)		–

Numbers are medians (interquartile range, IQR) or *n* (%)

*CIS* clinically isolated syndrome; *MS* multiple sclerosis (according to McDonald 2010 criteria); *OCB* oligoclonal band

While the conversion rate (by clinical or MRI signs) in CIS patients showing intrathecal OCBs (310/362) was 74 % (229/310), it was 44 % (23/52) in those CIS patients with negative OCBs. In patients without brain lesions at first attack and presence of intrathecal OCBs (30/44), conversion rate to MS was 60 % (18/30), whereas it was only 21 % (3/14) in those without OCBs (Fig. 1), revealing a positive predictive value of 79 % and a likelihood ratio for conversion of 3.4 in this subset of patients.

**Fig. 2:**
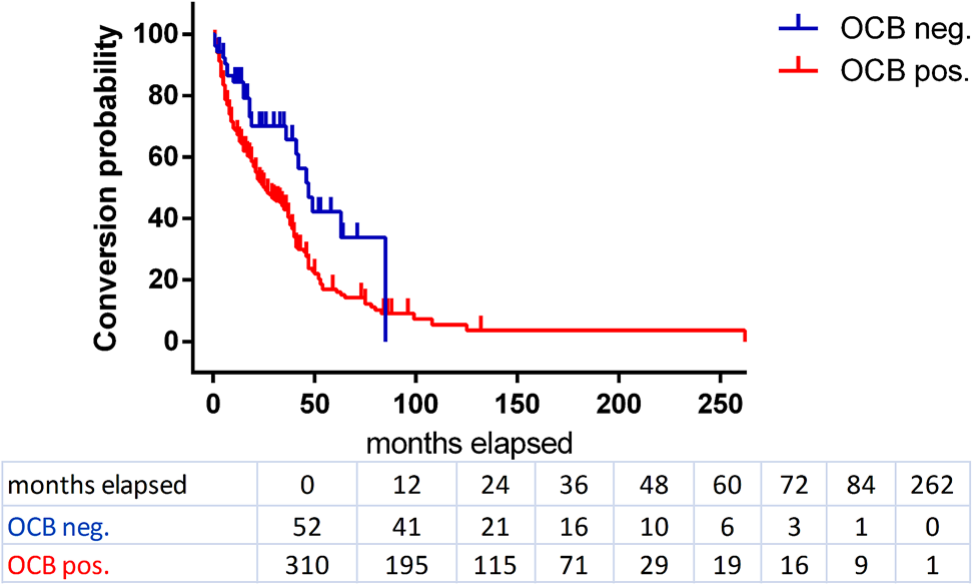
Kaplan–Meier survival curves for OCB-positive (*red*) and -negative (*blue*) CIS patients concerning the time of conversion to definite multiple sclerosis according to McDonald 2010 criteria. Patients who did not convert to definite MS and/or whose follow-up time was less than 24 months were censored (OCB pos. = 62, OCB neg. = 19, indicated by *dash* on the curve). The numbers of subjects at risk are given in the table under the graph

The median conversion time to definite MS for CIS patients with positive OCBs was 25 months (95 % CI 21–34) compared to 47 months (95 % CI 36–85) in those patients without OCBs (Fig. 2). CIS patients with intrathecal OCBs were twice as likely to convert to definite MS as OCB-negative individuals (hazard ratio = 2.1, *p* = 0.0014).

In Table 2, sensitivity, specificity, positive (PPV) and negative (NPV) predictive values of MRI and CSF parameters concerning conversion from CIS to definite MS are summarized. Whereas OCBs show the highest sensitivity of 91 % and an NPV of 39 %, Montalban criteria and OCB yield the highest specificity of 95 % (same result is achieved with Montalban criteria alone) and Montalban criteria reveal the best PPV of 92 %.

**Table 2:** Sensitivity, specificity, positive (PPV) and negative (NPV) predictive values in percent (95 % confidence intervals) for CSF and MRI parameters regarding conversion of clinically isolated syndrome to definite multiple sclerosis

Parameter	Sensitivity	Specificity	PPV	NPV
Barkhof	24 (18.5–29.9)	80 (70.4–90.3)	81	23
Swanton	27 (21.5–33.3)	79 (69.9–89.2)	82	24
Montalban	15 (10.3–19.8)	95 (89.8–100)	92	24
cell count (>4/µl)	58 (51.3–64.5)	55 (42.4–67.2)	82	27
OCB	91 (87.1–94.7)	21 (10.8–31.1)	80	39
Cell count and OCB	57 (50.3–63.5)	58 (45.8–70.3)	83	28
Barkhof and OCB	21 (16.0–26.9)	82 (72.7–91.8)	81	23
Swanton and OCB	24 (18.1–29.4)	81 (70.8–90.5)	81	23
Montalban and OCB	13 (8.8–17.7)	95 (89.8–100)	91	24

Values are given for the comparison between the absence or presence of the regarding condition (e.g., OCBs-positive subjects compared to OCB-negative). Follow-up for non-converters had to be at least 24 months

Among the 44 MS patients satisfying the McDonald 2010 criteria at disease onset, follow-up disease activity (by clinical or MRI signs) was observed in 36 out of those 41 who were OCB positive (88 %), whereas only one of the three patients without OCBs developed a second clinical attack detected by clinical or MRI signs (Fig. 1).

## Discussion

The last revision of the McDonald diagnostic criteria for MS, dating from 2010, does not include CSF criteria for a diagnosis of RRMS, while OCBs may support a diagnosis of PPMS [2]. Nevertheless, studies according to the previous diagnostic criteria showed that OCB positivity in CIS patients is a predictor for conversion to CDMS in adults [11, 15, 20] and children [16] independent of other factors [15, 21]. We now investigated the prevalence and predictive value of OCBs in the revised McDonald criteria 2010 era. Only 11 % of our patients with a first manifestation suggestive of MS met the revised McDonald criteria at disease onset. Thus, for the majority of CIS patients, further information allowing estimating the risk to develop definite MS would be of (high) value. We found OCB positivity in 86 % of 362 CIS patients at clinical onset. Those patients were approximately twice as likely to convert to definite MS and within a shorter period of time as OCB-negative CIS patients. This is in concordance with results in other cohorts, mostly referring to the revised McDonald criteria 2005 [11, 15, 21]. Whereas MRI criteria (Barkhof, Swanton, and Montalban) showed a higher specificity, OCBs performed best concerning sensitivity and NPV. Combining MRI criteria and OCB did not add to sensitivity or specificity for the prediction of conversion to definite MS compared to MRI criteria alone, despite that both MRI and the presence of OCBs in CSF have been repeatedly shown to be independent predictive factors [15, 21]. In another subset of patients, i.e., those who do not show any brain lesions (11 % of all patients), OCB-positive individuals are three times more likely to develop definite MS than OCB-negative patients; hence OCBs are the only predictor of conversion in this subset of CIS patients.

Our data further underline the utility and importance of CSF diagnostics, especially the detection of OCBs. We thus continue to recommend the inclusion of OCBs in the diagnostic workup of patients under the differential diagnosis of an MS [3]. CIS patients showing a positive OCB finding are at a higher risk of developing a definite MS; particularly in CIS patients not showing lesions, OCBs are of great interest. This might be helpful for the clinician to decide whether or not a disease-modifying immunotherapy should be started.
